# Alcohol Use As a Risk Factor in Infections and Healing: A Clinician’s Perspective

**DOI:** 10.35946/arcr.v37.2.03

**Published:** 2015

**Authors:** Giraldina Trevejo-Nunez, Jay K. Kolls, Marjolein de Wit

**Affiliations:** Giraldina Trevejo-Nunez, M.D., is an instructor in medicine and Jay K. Kolls, M.D., is the director of the Richard King Mellon Foundation Institute for Pediatric Research and professor of pediatrics, both at the University of Pittsburgh School of Medicine. Dr. Kolls also is vice chair for translational research, Department of Pediatrics, Children’s Hospital of Pittsburgh at the University of Pittsburgh Medical Center, University of Pittsburgh, Pittsburgh, Pennsylvania. Marjolein de Wit, M.D., is an associate professor of medicine in the Division of Pulmonary Disease and Critical Care Medicine, Department of Internal Medicine, Virginia Commonwealth University, Richmond, Virginia.

**Keywords:** Alcohol use, alcohol use and misuse, alcoholism, alcoholics, infection, immunity, immune system, pneumonia, molecular mechanisms, secondary immune deficiency, gut, lower respiratory mucosal immunity

## Abstract

Physicians have recognized for more than a century that alcohol use is associated with infections and that alcoholics are especially at risk for pneumonia. Clear evidence now indicates that alcohol has a systemic effect on every organ. This review first presents a clinical case to describe a patient with immunity issues complicated by alcohol use—a setting familiar to many clinicians. This is followed by a description of the molecular mechanisms that explain the secondary immune deficiency produced by alcohol in the host, focusing mostly on the gut and lower respiratory mucosal immunity. The goal of this review is to increase awareness of the new mechanisms being investigated to understand how alcohol affects the human immune system and the development of new strategies to attenuate adverse outcomes in the affected population.

Alcohol use and misuse have been part of human society for centuries. Early physicians recognized since the 1800s that alcohol produced not only impairment of the senses but also higher predisposition for tuberculosis. William Osler, the father of scientific medicine, reported in 1905 that patients who misused alcohol had higher predisposition to pneumonia (Osler 2001).

Between 2006 and 2010 in the United States, excessive alcohol consumption resulted in approximately 88,000 deaths (data based on 11 U.S. States), and the median alcohol-attributable death rate was 28.5 per 100,000 population (Gonzales et al. 2014). Furthermore, the potential years of life lost attributed to alcohol (estimate of the average years people would have lived if they had not died prematurely) averaged 2.5 million years annually from 2006 through 2010 (Gonzales et al. 2014). Importantly, the majority of alcohol-related deaths and potential life lost were among working-age adults (20- to 64-year-olds). In addition, the estimated cost of excessive drinking was $223.5 billion in 2006, from which the majority represented loss of productivity, valued at $161.3 billion, followed by increased health care costs and criminal justice costs of $21 billion each (Bouchery et al. 2011).

Patients with a history of heavy acute or chronic alcohol use have higher rates of hospitalizations (Smothers and Yahr 2005), longer hospital stays (de Wit et al. 2011), risk for major complications when they also suffer polytrauma (like pneumonia, bleeding disorders, and withdrawal syndrome) (Spies et al. 1996), increased mortality (de Wit et al. 2011), higher intensive care unit admissions, and greater postoperative complications when they are admitted to hospitals and when they need surgery (Delgado-Rodriguez et al. 2003), compared with patients with no history of alcohol use. The following section illustrates the scope of these problems with a clinical case.

## Clinical Case

A 42-year-old white male presented to the emergency department with a 3-day history of fevers, shortness of breath, and a mucus-producing cough. He reported a history of hypertension, smoking half a pack to one pack of cigarettes per day, and drinking four to six beers per day. (Although not reported, the specific size of each beer is 22 ounces. Thus, the patient consumed 88 to 132 ounces of beer per day, an equivalent of approximately 7 to 11 standard drinks per day). He reported no history of lung disease, such as chronic obstructive pulmonary disease or asthma.

The patient received immediate attention as a result of having reduced oxygen supply. Oxyhemoglobin saturation, a measure of oxygen in the blood, was low (89 percent). His blood pressure was 102/72 mmHg and heart rate 118 beats per minute. This blood pressure was well below the patient’s baseline pressure. The patient appeared ill and had shortened speech pattern. No jugular venous distention was present. His cardiac examination was normal and the lung examination revealed dullness to percussion at the right base, along with bronchial breath sounds and increased resonance of vocal sounds (i.e., egophony) over the right lung base. The patient’s abdomen was non-distended and non-tender, he had normal bowel sounds, and his liver edge was 5 centimeters below the right costal margin. His extremities revealed no clubbing, skin discoloration as a result of low oxygen (i.e., cyanosis), or edema, and his neurological exam was remarkable for a patient who was alert and oriented to time, place, and location. The patient moved all his extremities spontaneously.

Laboratory data revealed elevated white blood cell count (i.e., leukocytosis) to 15,600 cells per microliter with 13 percent bands, toxic granulations, and mild low blood platelet count (i.e., thrombocytopenia) to 123,000 cells per microliter. Electrolytes were remarkable for low potassium levels (i.e., hypokalemia) to 3.2 millimoles per liter, which were repleted. Arterial blood gas was consistent with the buildup of carbon dioxide in the lungs (i.e., acute respiratory acidosis) and a low level of oxygen in the blood (i.e., hypoxemia). A chest X-ray revealed dense alveolar consolidation of the right lung base. The patient was found to have severely low blood pressure because of his ongoing infection and inflammatory response (i.e., septic shock) and overly active proteins that control blood clotting (i.e., disseminated intravascular coagulation) attributed to pneumonia.

The patient received supplemental oxygen and oxyhemoglobin saturation increased from 89 percent to 95 percent. Broad-spectrum antibiotics were initiated within 2 hours after the patient arrived at the emergency department. Despite administration of 2.5 liters of normal saline, the patient’s blood pressure further decreased to 92/54 mmHg. As a result, a central venous catheter was placed, and norepinephrine was given to raise the patient’s blood pressure. The patient was transferred from the emergency department to the medical intensive care unit. However, shortly after arrival to the medical intensive care unit, oxyhemoglobin saturation decreased to 86 percent, and the patient continued to have a difficult time breathing. He was emergently intubated with an endotracheal tube because of acute respiratory failure, and mechanical ventilation was initiated. A repeated chest X-ray revealed progression of air-space disease: It involved both lungs and all four quadrants. The patient was diagnosed with acute respiratory distress syndrome, and ventilator settings were adjusted to decrease tidal volume to 6 milliliters per kilogram ideal body weight. The patient also required increasing amounts of positive end expiratory pressure to 14 centimeters of water and fraction of inspired oxygen 70 percent. Over the ensuing 2 to 3 days, the patient’s blood flow improved slowly, his norepinephrine dose was decreased, and leukocytosis resolved. Blood cultures grew cephalosporine-sensitive *Klebsiella pneumoniae*.

Although the patient’s clinical condition improved, his neurocognitive function did not. He was given sedatives to facilitate mechanical ventilation. However, he required high doses of the depressant midazolam, and his mental status fluctuated from being unresponsive to sitting upright in bed attempting to remove the endotracheal tube. On hospital day 2, the patient developed delirium. The presence of the endotracheal tube prevented the patient from expressing himself verbally, and the medical team had very limited ability to communicate with him. The team was unable to determine whether the patient had a headache, nausea, or experienced hallucinations or the feeling of insects crawling on one’s skin (i.e., formication), signs of alcohol withdrawal. Rather, the medical team was limited to evaluating gross motor movements, behavior, and facial expressions. The patient’s fevers and rapid heart rate (i.e., tachycardia) persisted for days, and the physicians were unable to determine whether these were attributed to ongoing infection and inflammatory response (i.e., sepsis) or if the patient was developing superimposed alcohol withdrawal. Administration of midazolam was based on motor behaviors and a modified Clinical Institute Withdrawal Assessment for Alcohol (CIWA-Ar). Realizing its limitations in the non-verbal critically ill patient, physicians were left attempting different strategies for management of sedation. They increased the dose of midazolam, only to find that the patient became overly sedated and unresponsive. Empiric trials of opioids were attempted in order to determine if the patient was experiencing pain, but again this strategy did not seem to improve the patient’s neurocognitive function. Next, haloperidol, an antipsychotic, was administered to control agitation and perhaps aided in management of delirium. The team continued to attempt different strategies to manage his fluctuating agitation and sedation, including attempts to limit midazolam administration in favor of administration of haloperidol. However, after approximately 7 days, the patient was more alert and cooperative, but it was not clear what led to the improvement. He remained delirious but was able to follow commands. His septic shock resolved, and mechanical ventilation was removed.

## Discussion

This case illustrates the complexities of managing critically ill patients with alcohol use disorder (AUD), which is common in such patients and may occur in 40 percent of hospitalized patients. Critically ill patients with AUD have unique problems, including needs for higher doses of sedatives and increased risk of requiring mechanical ventilation (de Wit et al. 2007*a*). Patients with AUD are at increased risk of developing pneumonia and sepsis. In addition, pneumonia encompasses a larger geographic area of the lung in patients with AUD compared with patients without AUD (de Roux et al. 2006; Fernandez-Sola et al. 1995; Ruiz et al. 1999; Saitz et al. 1997; Torres et al. 1991). This patient population also has prolonged fevers and slower radiographic resolution of air-space disease. Furthermore, acute respiratory distress syndrome, which carries a mortality greater than 30 percent, is more common among patients with AUD who experience sepsis (Moss et al. 1996). Sepsis also is more severe and has a higher mortality in this patient population (O’Brien et al. 2007). Patients with AUD may not only experience higher severity of illness but also are at increased risk of developing neurocognitive complications, such as delirium. The patient in the case scenario developed agitation, which could have been a result of a variety of etiologies, including dyspnea, anxiety, pain (from the endotracheal tube, lying in bed, or from procedures), delirium induced by critical illness, alcohol withdrawal, or delirium tremens (de Wit et al. 2007*b*). Despite the high prevalence of these disorders among critically ill patients, only limited data are available to aid physicians in managing this patient cohort so as to optimize patient outcome (Awissi et al. 2013; Sarff and Gold 2010). For instance, the management of alcohol withdrawal has not changed much over the last two decades and is centered in supportive care (Sarff and Gold 2010), electrolyte replenishment, vitamin supplementation (especially thiamine), benzodiazepine administration, and consideration of alternative agents such as propofol and beta blockers. Benzodiazepines (usually midazolam and lorazepam) are routinely administered during the course of mechanical ventilation to treat agitation. However, benzodiazepines also are linked to the development of delirium, a condition associated with increased mortality (Ely et al. 2004).

## Effects of Alcohol on the Mucosal Immune System

For more than a century, physicians have noticed that alcohol produces abnormalities in the host defenses. As early as the late 1800s, scientists performed studies to determine how ethanol produced detrimental effects in humans. Human studies have provided important epidemiological data demonstrating an association between alcohol consumption and risk of infection. This increased risk of infection has been attributed to alcohol’s effect on the immune system. The following sections focus mainly on the effects of alcohol on gut, lung, and skin mucosal immunity.

### Effects on Mucosal Gut (Intestinal) Immunity

The intestinal mucosa plays an important role in alcohol metabolism, as the epithelial surface incorporates alcohol into the blood system by passive diffusion, which accounts for approximately 80 percent of alcohol absorption (Norberg et al. 2003). The other 20 percent is absorbed through the gastric mucosa.

A recent study described the effects of acute alcohol binge drinking on gut homeostasis in healthy human study participants (Bala et al. 2014). Higher levels of alcohol (about 90 mg/dl), which occurred 1 hour after oral ingestion of vodka (40 percent ethanol), correlated with higher expression of serum bacterial DNA as measured by the abundance of 16S rDNA (a conserved genetic component of several bacteria that should not be present in the blood in healthy people). Furthermore, endotoxin, which is produced by Gram-negative organisms, was increased in serum as early as 30 minutes after alcohol ingestion and persisted at high levels for 3 hours after ingestion. These data suggested that even acute episodes of alcohol intoxication provoked abnormalities in the gut epithelium. These findings are supported by studies performed in rats, which showed evidence of a mild increase of endotoxemia after one dose of acute alcohol ingestion (Rivera et al. 1998). Thus, it is critical to understand not only how chronic alcohol consumption increases endotoxin levels in the host but also how acute alcohol intoxication affects translocation of bacteria or microbial products. One known mechanism involves alcohol’s role in increasing permeability of the intestine, leading to bacterial translocation and higher levels of the molecules found in the outer membrane of Gram-negative bacteria (i.e, lipopolysaccharide) in the blood (Bode and Bode 2005; Schaffert et al. 2009; Szabo and Bala 2010).

The exact mechanisms underlying alcohol’s role in increasing gut permeability and/or transient endotoxemia are not clearly elucidated, but recent studies have suggested some possible causes (see figure). Cytochrome P4502E1 is an enzyme present in the liver and is involved in the metabolism and oxidation of alcohol, fatty acids, and foreign compounds (Cederbaum 2010). CYP2E1 is the most highly expressed isoform of the CYP450 cytochrome enzymes and is highly expressed not only in the liver but also in the small intestine and colon. In addition, an acute model of alcohol binge intoxication in mice shows that alcohol can induce the expression of intestinal CYP2E1 and that this induction is correlated with higher endotoxemia and translocation of liver bacteria. These outcome measures were substantially reduced, however, in Cyp2e1^−^ null mice (genetically modified mouse in which Cyp2e1 gene is inactive) compared with wild-type control mice, suggesting that CYP2E1 is essential for development of gut leakiness. The deleterious effects of alcohol-induced CYP2E1 were ameliorated with treatment with the antioxidant N-acetylcysteine (Abdelmegeed et al. 2013).

Activated CYP2E1 can produce oxidative stress and tissue damage as a result of reactive oxygen species, and this oxidative stress can damage intestinal barrier function. In vitro studies have shown that acute exposure for 2 to 4 hours of Caco-2 cells (human colon adenocarcinoma cell line) to 43 mM of alcohol increased CYP2E1, which correlated with higher intestinal permeability (measured by trans-electrical epithelial resistance). Reducing the expression of CYP2E1 (i.e., knockdown) by small-interfering RNA reversed alcohol-induced alteration in cell permeability (Forsyth et al. 2013). Furthermore, CYP2E1 metabolism of alcohol and its oxidative stress products induced reduction and oxidation-sensitive circadian CLOCK (clock circadian regulator) and PER2 (period circadian clock 2) protein expression in intestinal epithelial cells (Caco-2 cells). The induction of CLOCK and PER2 promoted intestinal hyperpermeability. Thus, alcohol can disrupt circadian rhythms at the level of gene transcription in the intestine. Circadian clock genes are those that depend on day and light and feeding patterns. Growing evidence suggests that alcohol disrupts circadian rhythmicity, probably by intestinal-derived lipopolysaccharide (Voigt et al. 2013). Inhibition of CYP2E1 protein expression stopped intestinal hyperpermeability as well as induction of CLOCK and PER2 (Forsyth et al. 2013) and knocking out Clock or Per2 in intestinal epithelial cells also ameliorated alcohol-induced intestinal hyperpermeability (Swanson et al. 2011).

In the case of chronic ethanol models, the disruption of intestinal permeability is highly correlated with the development of alcoholic liver disease (Mathurin et al. 2000; Szabo 2015; Wang et al. 2014). That is, bacterial translocation causes high endotoxin levels in the circulation, which induces the production of cells that regulate inflammation (i.e., tumor necrosis factor-alpha [TNF-α]) by Kupffer cells in the liver. This chain of events can then lead to alcoholic liver disease (Bode and Bode 2005). Furthermore, bacterial overgrowth in the small intestine probably contributes to higher endotoxin blood levels (Fouts et al. 2012). One other possible mechanism of increased endotoxin levels upon alcohol intake is through the upregulation of microRNAs, which are small non-coding RNAs involved in modulating protein synthesis, likely by inhibiting translation of mRNA (the coding RNA). Most tissues produce specific microRNAs, and an increasing amount of research over the last 10 years supports their role in protein expression and regulation. That said, research also shows that microRNAs are affected by alcohol intake. Thus, high levels of specific microRNAs are associated with gut leakiness. In particular, the microRNA miR-212 inhibits gene expression of zonula occludens gene (ZO-1), an important gene that regulates the formation of tight junctions in the gut lumen, especially in the colon (Tang et al. 2008). Furthermore, chronic alcohol intake increases tissue-specific microRNA miR-155, which contributes to alcohol-induced small bowel inflammation and alteration of gut barrier integrity in mouse small intestine. This has been shown by research in which miR-155 knockout mice, despite chronic alcohol administration, were protected from higher serum endotoxin levels and preserved their regenerating islet-derived protein III beta (Reg3b) protein expression, an antimicrobial peptide that plays a role in intestinal barrier integrity, unlike wild-type mice (Lippai et al. 2014). In addition, research using a non-human primate model of chronic alcohol consumption (Asquith et al. 2014) found that expression of miR-155 was correlated with alcohol administration. The increase in miR-155 among alcohol-exposed primates was inversely correlated with the production of immune cell-signaling proteins (i.e., cytokines) by colonic T cells.

Research with a chronic alcohol model in rhesus macaques has found a detrimental effect on immune cells in the gut. The alcohol-exposed group had a smaller percentage of immunity-boosting TNF-α^+^ cluster of differentiation 8 (CD8^+^) cells in the layer of intestinal tissue beneath the epithelium (i.e., lamina propria) compared with non–alcohol-exposed animals (Asquith et al. 2014). The study also found that alcohol-exposed animals had a decreased percentage of intraepithelial immune cells known as IL17^+^ INFγ^+^ CD4^+^ T cells from the jejunum and a decreased percentage of IL17^+^ IFNγ^+^ CD8^+^ T cells from the lamina propria in the ileum. Alcohol also has been associated with the profound loss of gut-associated lymphoid tissues in a mouse model of Salmonella typhimurium infection (Sibley and Jerrells 2000). Both mesenteric lymph nodes and Peyer’s patches lymphocytes, important immune system components, were decreased from day 3 to day 7 in the alcohol-fed mice group, compared with the pair-fed and chow controls. The alcohol-fed mice exhibited higher bacteria levels in the liver and intestinal tract compared with pair-fed control animals (Sibley and Jerrells 2000). Thus, this body of evidence shows the deleterious effects and mechanism of action by which alcohol perturbs mucosal gut immunity.

## Effects on Mucosal Lung Immunity

The effects of alcohol intake undermine immune defenses in both the upper and lower airways. This review will focus mainly on mucosal lung immunity of the lower airways. The alveolar macrophage is an important immune cell affected by alcohol consumption. Alveolar macrophages have the receptor for the granulocyte-macrophage colony-stimulating factor (GM-CSF), which is important for terminal differentiation of fetal monocytes in the lung into mature alveolar macrophages (Guilliams et al. 2013; Schneider et al. 2014). Interaction of GM-CSF with its receptor leads to the nuclear binding of the transcription factor PU.1, which is important for alveolar macrophage gene regulation and development (Bonfield et al. 2003). In chronic alcohol-fed rats, the alveolar macrophage displayed decreased membrane expression of the GM-CSF receptor as well as impaired bacterial ingestion (i.e., phagocytic activity) (Joshi et al. 2005). These effects were reversed with recombinant GM-CSF, which restores GM-CSF signal responsiveness and innate function in alveolar macrophages of alcohol-fed rats.

Moreover, alcohol activates nicotinamide adenine dinucleotide phosphate (NADPH) oxidases upon oxidative stress in the alveolar macrophages. When activated, these enzymes can produce reactive oxygen species (i.e, superoxide, hydrogen peroxide). Specifically, chronic alcohol intake increases the level of NADPH oxidase 1 (Nox1), NADPH oxidase 2 (Nox2), and NADPH oxidase 4 (Nox4) at the transcriptional and protein levels in alveolar macrophages of animal models and human alveolar macrophages from alcoholic study participants (Yeligar et al. 2012). In addition to increased oxidative stress, chronic alcohol use results in the depletion of important alveolar antioxidants like glutathione or its precursors. In one study, restoration of glutathione through its precursor N-acetylcysteine into the alveolar environment improved alveolar phagocytic function and decreased alveolar damage in animal models (Yeligar et al. 2014).

Zinc is another important element impaired by alcohol intake. Joshi and colleagues (2009) found that alcohol-fed animals had lower zinc levels in the alveolar compartment compared with control animals and that this level did not correlate with zinc blood levels, which were within normal range. A study performed in human study participants corroborated the finding that alveolar macrophages from alcoholics had lower intracellular zinc levels than nonalcoholic study participants (Mehta et al. 2013). Furthermore, these human macrophages had decreased phagocytic activity when exposed to *Staphylococcus aureus* in vitro. Intracellular zinc levels and phagocytic activity were improved when alveolar macrophages were treated with Zinc and procysteine, a glutathione precursor (Mehta et al. 2013).

Alcohol also has been found to affect lung immunity through other mechanisms. These include alterations in the recruitment of white blood cells (i.e., neutrophils) into the alveolar space, impairment of neutrophil movement in response to infection, and decreased activation of proteins that induce an immune response (Boe et al. 2001, 2003). These findings have been observed in rodent models of acute alcohol intoxication with *S. pneumoniae* and *K. pneumoniae* lung infection (Boe et al. 2001; Quinton et al. 2005). Further investigation into the impaired recruitment of neutrophils to the alcoholic lung upon infection has revealed that alcohol enhances the phosphorylation of the transcription factor signal transducer and activator of transcription 3 (STAT3) in nucleated bone marrow cells, blunting hematopoietic precursor cell response (i.e., formation of immune cells) (Siggins et al. 2011) against pneumococcal infection in a mouse model of acute chronic alcohol intake.

Furthermore, alcohol seems to produce abnormalities and decreased numbers in natural killer (NK) cells, which are decreased in mouse models of alcohol consumption (Blank et al. 1993). In research with a mouse model, Zhang and Meadows (2008) reported that chronic alcohol impaired the release of NK cells from the bone marrow, which translates into decreased bone marrow–derived NK cells in the spleen and higher percentages of thymus-derived NK cells (Zhang and Meadows 2008). The alcohol-induced imbalance of NK cells may be disadvantageous for the host because thymus-derived NK cells have less cytolytic capacity and more cytokine production properties. The observation that alcoholic patients have predisposition to viral infections like cytomegalovirus (Arase et al. 2002; Bekiaris et al. 2008) and influenza as well as certain tumors may be related to NK-cell dysfunction. In a mouse model of chronic alcohol intake, the populations of NK cells in the spleen were decreased at 1 week compared with controls, which accounted for decreased cytotoxic activity. This difference was attributed to decreased percentage and decreased absolute number of the NK T cells NK1.1^+^ and CD3^−^ negative cells (marker of NK T cells). However, the groups did not differ in number or percentages at 8 weeks post–alcohol intake. A decrease in the NK subtype Ly49H^+^, CD11b^+^, CD27^−^ was observed 10 weeks after alcohol consumption. This subtype has been involved with predisposition to cytomegalovirus infections in a mouse model. Thus, it seems that alcohol may affect selective populations of NK cells in a time-dependent manner (Ballas et al. 2012).

## Effects on Mucosal Skin Immunity

Like any other organ in the human body, the skin is also affected by alcohol intake. Alcoholism is associated with higher rates of wound infection and delay in wound closure. It is associated with increased risk for *Staphylococcus aureus* infection, including methicillin-resistant *Staphylococcus aureus*, *Streptococcus pyogenes,* and *Vibrium vulnificus*.

Ethanol seems to impair dermal fibroblast function, which plays a role in wound healing. Dermal fibroblasts display proliferative responses along with secretion of growth factors. In vitro studies of human fibroblasts exposed to alcohol demonstrated a reduction in dermal wound breaking strength (immature wound) (Ranzer et al. 2011). Although human skin differs in cellular components compared with other mammalian species, mouse models of skin infection and alcohol consumption have helped researchers understand alcohol’s damaging effects on the skin. One study found that mice had 30 to 50 percent fewer epidermal immune cells (i.e., Langerhans cells) after 4 weeks of chronic alcohol consumption (Ness et al. 2008). This effect is likely to account for decreased immune surveillance once the host encounters a pathogenic organism in the skin.

In the mouse epidermis, a type of resident skin T cell known as dendritic epidermal T cells (DETCs) are prompt to respond to skin injury, participate in wound healing (Jameson et al. 2002), and fight against tumor formation. These resident T cells have a gamma delta T-cell receptor (γδ TCR) and do not need antigen presentation or major histocompatibility complex (MHC) class molecules to mature to have an effector function. In the mouse, DETCs are exclusively restricted to the epidermis and are absent in other tissues, peripheral circulation, or lymph nodes. DETCs also display receptors and molecules (e.g., junctional adhesion molecule-like [JAML] protein, NK group 2, member D [NKG2D], cluster of differentiation 69 [CD69]) to facilitate their crosstalk with other cells in the network upon skin stress or damage. Inhibition of JAML leads to decreased γδ T-cell induction and delayed wound healing (Witherden et al. 2010).

Chronic ethanol intake can also affect skin T cells in mouse models. DETCs are significantly decreased in ethanol-fed mice compared with water-fed controls and ethanol-fed mice show significant depletion of dermal γδ T cells compared with controls. Furthermore, dermal γδ T17 cells have decreased interleukin-17 production following administration of the immunosuppressive drug anti-CD3 monoclonal antibody (Parlet and Schlueter 2013). So it seems that skin T-cell populations are affected by ethanol and that T cells that express the γδTCR are more affected, whereas those that express the αβTCR seem to be unaffected.

Some differences between human and mouse skin need to be considered, however. For instance, DETCs are only found in the mouse epidermis, in which they represent 98 percent of CD3^+^ T cells. In the human epidermis, by contrast, αβ^+^ and γδ^+^ T cells (mostly Vδ1, a subset of gamma delta cells) are represented equally. Nevertheless, the mechanisms studied in the mouse models bring up questions that can be studied in human cells and these studies may help to reveal novel pathways by which ethanol impairs human skin immunity.

As illustrated above, patient care is clearly complicated by alcohol-induced immunity issues. The mechanisms described explain alcohol’s role in causing immune deficiency in the gut and respiratory mucosa. With greater awareness of these mechanisms, researchers and clinicians will have a better understanding of how alcohol affects the human immune system, leading to the eventual development of new strategies to reduce adverse outcomes in the affected population.

## Figures and Tables

**Figure:**
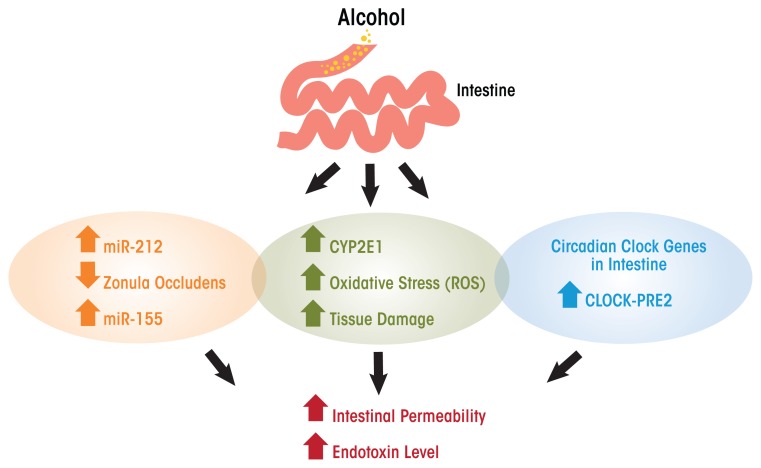
Alcohol use increases intestinal permeability and endotoxin levels. The mechanisms include increases in microRNA miR-212 levels, which decrease gene expression within the zonula occludens, resulting in increases to miR-155 that produce intestinal inflammation. Alcohol induces expression of the enzyme CYP2E1, increasing reactive oxygen species, which damage tissue through increases in oxidative stress. Alcohol also increases the expression of circadian clock genes that alter intestinal permeability.
